# Evaluation of Cell Proliferation and Wound Healing Effects of Vitamin A Palmitate-Loaded PLGA/Chitosan-Coated PLGA Nanoparticles: Preparation, Characterization, Release, and Release Kinetics

**DOI:** 10.1021/acsomega.2c07232

**Published:** 2023-01-06

**Authors:** Lala Baghirova, Elif Kaya Tilki, A. Alper Öztürk

**Affiliations:** † Graduate School of Health Sciences, Faculty of Pharmacy, Department of Cosmetology, Anadolu University, 26470Eskişehir, Turkey; ‡ Faculty of Pharmacy, Department of Pharmacology, Anadolu University, 26470Eskişehir, Turkey; § Faculty of Pharmacy, Department of Pharmaceutical Technology, Anadolu University, 26470Eskişehir, Turkey

## Abstract

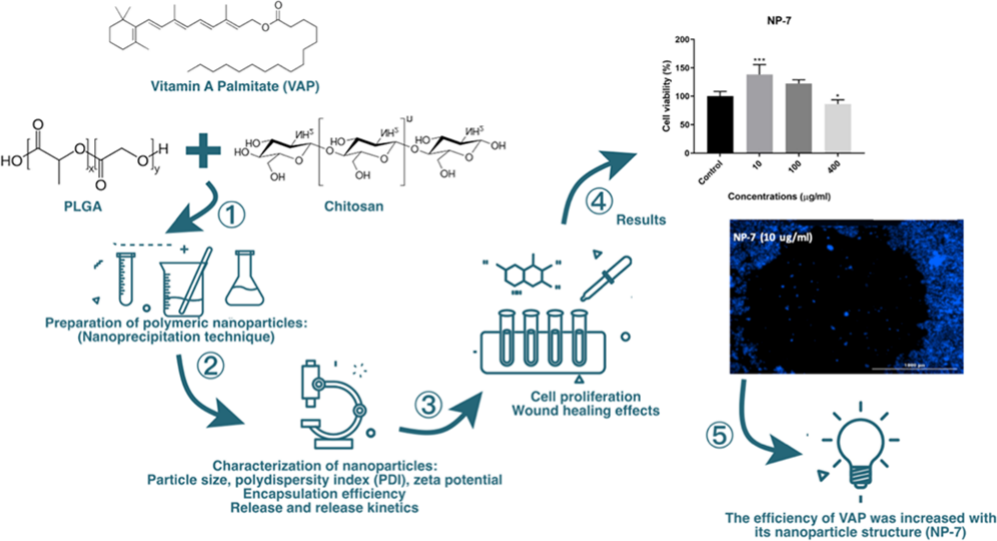

In this study, vitamin A palmitate (VAP)-loaded poly(lactic-*co*-glycolic acid) (PLGA)/chitosan-coated PLGA nanoparticle (NP) systems were prepared by the nanoprecipitation technique. The prepared systems were characterized by parameters such as particle size, polydispersity index (PDI), ζ-potential, encapsulation efficiency, *in vitro* dissolution, and release kinetic study. Then, the cytotoxicity and wound healing profiles of the designed NP formulations in HaCaT (human keratinocyte skin cell lines) were determined. The particle size of VAP-loaded NPs was obtained between 196.33 ± 0.65 and 669.23 ± 5.49 nm. PDI data proved that all NPs were prepared as high quality and monodisperse. While negative ζ-potential values of Blank-NP-1 and NP-1 encoded PLGA NP formulations were obtained, positive ζ-potential was obtained in chitosan-coated NPs. *In vitro* release studies of NPs observed rapid dissolution in the first 1–6 h, but prolonged dissolution of VAP after rapid dissolution. As a result of cell culture studies and wound healing activity studies, it was determined that NP-7 was the most effective. It was thought that the reason for this was that the NP-7 coded formulation was a chitosan-coated PLGA nanoparticle with the smallest particle size, and it was concluded that the efficiency of VAP was increased with its nanoparticle structure. This study demonstrated the similar wound healing effects of VAP-loaded nanoparticle systems, in particular NP-7, which increases keratinocyte cell proliferation at lower concentrations (10 μg·mL^–1^) than vitamin A alone (100 μg·mL^–1^). VAP-loaded nanocarriers that can be used in the pharmaceutical industry have been successfully produced and the results obtained have been evaluated as promising for this industry.

## Introduction

In addition to being time-consuming and expensive, the process of developing a novel medicinal chemical frequently fails. However, utilizing a variety of strategies to boost their bioavailability, targeting, efficacy, or safety may be a more effective method to use these drugs in the clinic. The researchers have thoroughly investigated a number of strategies, including drug conjugates, therapeutic drug monitoring, targeted medication therapy, and nanoparticle (NP)-based drug delivery systems.^1,2^ Besides, there are different approaches. Examples of these are conceptually innovative strategies to exploit ferroptosis against tumors, bidrug nanoplatforms, and drug carrier-free photodynamic nanodrugs to enable the regulation of dendritic cells.^3−5^ With systems like NPs, high-dose therapies with conventional pharmaceuticals can be administered at lower doses, and high effects can be produced at low doses.^6−8^ Nowadays, there are many different uses for NP-based therapeutics, and advancements in nanotechnology provide fresh approaches to tackling medical issues. Recently, novel materials in the nanoscale range have been developed quickly by nanodelivery systems. These materials are used to deliver therapeutic medicines to specific targeted areas in a regulated manner. It has offered several intriguing therapeutic opportunities, and several products are already available.^9^ The gaps in the therapy with nanoparticle systems are tried to be filled in many cases, such as cancer,^10−13^ disorders brought on by microorganisms,^14,15^ pain,^16−19^ oxidative-induced diseases,^20,21^ and Covid-19,^21^ and treatment with nanoparticles is a successful course.

The fat-soluble polyunsaturated hydrocarbon vitamin known as vitamin A contains both retinoids and carotenoids.^22^ While vitamin A from animal sources is already in the form of retinol, which your body can readily absorb, vitamin A from plant sources is a carotenoid that needs to be converted by your body into retinol.^23^ Available in dry or oily forms, vitamin A palmitate (VAP) is the ester of retinol and palmitic acid.^24^ A fat-soluble vitamin called VAP is essential for the development and upkeep of healthy mucous membranes, skin, and hair. It has the power to improve skin suppleness, lessen skin roughness, and stop skin lipids from oxidizing.^25^ It functions by removing the top layer of skin, which accelerates cell turnover and gives the skin a smoother, younger, and fresher appearance. It enhances skin hydration, promotes cellular renewal, and slows the aging process. On the skin, topical vitamin A functions as an antioxidant. It stops the collagen loss and tissue shrinkage that typically accompany aging. In skin that has been harmed by the sun, vitamin A helps to minimize keratoses and restore normal, supple skin.^25^ The use of VAP for cosmetic/dermacosmetic purposes is quite common.^26−28^ A recent use for VAP is its use in wound healing.^29^

The skin functions as a protective barrier against physical damage, fluid loss, and the invasion of toxic substances.^30,31^ Cutaneous wounds are physical injuries that cause the skin to open or break, resulting in disruptions in normal skin anatomy and function.^32,33^ When the skin is injured, platelets initiate a hemostatic reaction to prevent blood loss from the wound. This reaction is characterized by vascular narrowing, platelet aggregation and degranulation, coagulation, and finally the formation of a fibrin clot. This clot also induces the migration of inflammatory cells to the damaged area. After this stage, the wound healing process proceeds through three major sequential pathways, including an inflammation phase, the formation of a cell proliferation/granulation tissue phase, and a remodeling/scar formation phase, with all of the events requiring the interaction of many cell types.^34,35^ Fibroblasts are ubiquitous mesenchymal cells that synthesize collagen and other matrix macromolecules for the structural protection of connective tissues. Collagen I is one of the dermal ECM proteins excreted by transforming dermal fibroblasts activated by growth factor-β (TGF-β), a multifunctional growth factor that regulates the expression, accumulation, and transformation of extracellular matrix proteins in the skin.^36^ The systemic organization of the tissue is crucial for wound healing as it is vital to its integrity and strength.

Poly(lactic-*co*-glycolic acid) (PLGA) was used as the polymer, chitosan was used as the coating material, and the nanoprecipitation method was preferred to produce nanoparticles. PLGA nanocarriers encapsulating drugs such as antibiotics, anti-inflammatory drugs, proteins/peptides, and nucleic acids targeting various stages/signal loops of wound healing have provided optimum results in the literature.^37^ At the same time, PLGA is an FDA-approved polymer.^38^ One of the natural polymers commonly used in NP production and coating is chitosan. Chitosan is the most important derivative of chitin, produced by removing the acetate part from chitin. Chitosan has been widely used in pharmaceutical and medical areas because of its favorable biological properties such as safety, biocompatibility, biodegradability, low toxicity, bacteriostatic, fungistatic, hemostatic, anticholesterolemic, and anticancer properties.^39^ Because of these reasons, PLGA and chitosan were preferred in this study. Several approaches can be used to manufacture NPs.^38^ One of the two methods commonly used in the preparation of drug-loaded NPs is the double emulsion solvent diffusion/evaporation technique, whereas the other is the nanoprecipitation technique. The difference between these techniques is that hydrophilic drugs are loaded into NPs with the double emulsion solvent diffusion/evaporation technique, whereas hydrophobic drugs are loaded into NPs with the nanoprecipitation technique.^15^ Due to the low solubility of VAP in water, the nanoprecipitation method was used in this study.

Vitamin A palmitate (VAP)-loaded poly(lactic-*co*-glycolic acid) (PLGA)-based and VAP-loaded chitosan-coated PLGA-based nanoparticle (NP) systems were prepared by the nanoprecipitation technique. When all analyses were examined, nanoparticle formulations were prepared successfully, especially the chitosan-coated NP-7 coded formulation showed high efficiency and high wound healing potential at a low VAP dose. VAP is crucial to the wound healing process at every stage. It is well known for its capacity to promote epithelialization, fibroplasia, angiogenesis, fibroblasts, collagen synthesis, granulation tissue, and epithelial development. This study demonstrated the similar wound healing effects of VAP-loaded nanoparticle systems, in particular NP-7, which increases keratinocyte cell proliferation at lower concentrations (10 μg·mL^–1^) than vitamin A alone (100 μg·mL^–1^). Achieving a high pharmacological effect with low-dose treatment is very important both in terms of preventing side effects and in terms of economy, as it will reduce the budget allocated by countries for drugs. VAP-loaded nanocarriers that can be used in the pharmaceutical industry have been successfully produced and the results obtained have been evaluated as promising for this industry.

## Experimental Section

### Materials

Vitamin A palmitate (VAP) was a kind gift of Dermoskin (İstanbul, Turkey). Acetic acid, Dulbecco’s modified eagle’s medium (DMEM), fetal bovine serum (FBS), l-glutamine, potassium phosphate monobasic, and sodium hydroxide were purchased from Sigma-Aldrich (Germany). Acetone, dimethyl sulfoxide (DMSO), and Tween 80 were purchased from Merck (Germany). Ethanol, immunofluorescent dye (Hoechst 33258), chitosan (LMW), penicillin/streptomycin, Pluronic F-68, and Trypan blue were obtained from Carlo Erba (France), Cell Signaling Technology (The Netherlands), Sigma-Aldrich (Iceland), Gibco, Alfa Aesar (Germany), and Roche (Germany), respectively. Resomer RG 502 H (PLGA) and Span 60 were purchased from Sigma-Aldrich. All other chemicals and reagents used were of pharmaceutical and analytical grade.

### Preparation of Polymeric Nanoparticles

PLGA-based NPs were prepared by following the nanoprecipitation technique with some modifications.^7,40,41^ Briefly, a weighed amount of PLGA (60 mg) was dissolved in 3 mL of acetone together with Span 60 (32 mg). Three milliliters of this solution was added dropwise at a rate of 5 mL·h^–1^ into 10 mL of an aqueous solution under magnetic stirring. Acetone was then allowed to evaporate at room temperature under magnetic stirring for 4 h. The resulting aqueous dispersion was centrifuged to collect the NPs (11 000 rpm, 45 min, 4 °C) (Rotina-420R, Hettich Zentrifugen, Germany). After the NPs were collected, 5 mL of distilled water was added to wash the particles. The NPs dispersed in water were again subjected to the above-mentioned centrifugation process. This process was repeated twice to wash the NPs.

For VAP-loaded PLGA-based NP preparation, briefly, the procedure started by adding 6 mg of VAP to the organic phase solution (Table 1). Then, 3 mL of such solution with the drug was added dropwise at a rate of 5 mL·h^–1^ into 10 mL of an aqueous solution under magnetic stirring. Acetone was then allowed to evaporate at room temperature under magnetic stirring for 4 h. The resulting aqueous dispersion was centrifuged to collect the NPs (11 000 rpm, 45 min, 4 °C) (Rotina-420R, Hettich Zentrifugen, Germany). This process was repeated twice to wash the NPs.

**Table 1:** Formulation Ingredients^a^

	organic phase solution content				
code	PLGA (mg)	Span 60 (mg)	VAP (mg)	ACN (mL)	aqueous phase solution content (w/v)
Blank-NP-1	60	20	-	3	10 mL, 0.5% PF68 solution
NP-1	60	20	6	3	10 mL, 0.5% PF68 solution
Blank-NP-2	60	20	-	3	10 mL, 0.2% CS solution
NP-2	60	20	6	3	10 mL, 0.2% CS solution
blank-NP-3	60	20	-	3	10 mL, 0.2% CS solution containing 0.5% PF68
NP-3	60	20	6	3	10 mL, 0.2% CS solution containing 0.5% PF68
blank-NP-4	60	20	-	3	10 mL, 0.1% CS solution containing 0.5% PF68
NP-4	60	20	6	3	10 mL, 0.1% CS solution containing 0.5% PF68
blank-NP-5	60	20	-	3	10 mL, 0.05% CS solution containing 0.5% PF68
NP-5	60	20	6	3	10 mL, 0.05% CS solution containing 0.5% PF68
blank-NP-6	60	20	-	3	10 mL, 0.025% CS solution containing 0.5% PF68
NP-6	60	20	6	3	10 mL, 0.025% CS solution containing 0.5% PF68
blank-NP-7	60	20	-	3	10 mL, 0.0125% CS solution containing 0.5% PF68
NP-7	60	20	6	3	10 mL, 0.0125% CS solution containing 0.5% PF68

a PLGA: Resomer RG 502 H, VAP: vitamin A palmitate, ACN: acetone, PF68: Pluronic F-68, and CS: chitosan.

The above procedure was applied with minor modifications when preparing CS-coated formulations.^42−44^ In the CS-coated formulations, the aqueous phase consisted of CS solution and Pluronic F-68, both prepared in 2% acetic acid (v/v). The reason for using acetic acid in the study is the ability of chitosan to dissolve in acidic solutions.^45^ All remaining procedures are the same as above, and the formulation ingredients are presented in Table 1.

### Characterization of Nanoparticles

#### Particle Size, Polydispersity Index (PDI), and ζ-Potential

The particle size (PS) and polydispersity index (PDI) of NPs were measured using the dynamic light scattering technique on the Zetasizer Nano (Zetasizer Nano ZS, Malvern Instruments, Malvern, U.K.). PS and PDI of NPs prepared were measured by dispersing the formulation in distilled water. ζ-potential (ZP) was determined using the same instrument in a disposable folded capillary zeta cell at 25 °C room temperature and diluted with distilled water. For statistical analysis, all samples were measured in triplicate, and the average values and standard deviation of the measurements were calculated.

#### Encapsulation Efficiency (EE, %)

One milliliter of ethanol/acetone (1:1) was added to 5 mg of lyophilized nanoparticle formulation and then sonicated with a probe sonicator for 2 min to break up the nanoparticle structure. After the probe sonication process, it was centrifuged at 11 000 rpm for 5 min, the upper transparent part was removed, and the sample was filtered through a polyamide filter and analyzed at 330 nm in the UV spectrophotometer after necessary dilutions.

As a result of all of these experiments, the encapsulation efficiency was calculated with the help of eq 1 given below.
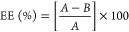
1where *A* is the theoretical amount of active substance in the 100% VAP amount added to the organic phase in the first stage of formulation preparation and *B* is the amount of active substance encapsulated.

### *In Vitro* Release

The *in vitro* dissolution studies of formulation were performed using the dialysis bag diffusion technique equipped with a magnetic stirrer (IKA Labortechnik RT 15 S000, Germany) at a speed of 100 rpm. Briefly, 5 mg of VAP and NP containing VAP equivalent to 5 mg of VAP was suspended in 1 mL of phosphate-buffer saline (PBS, pH 6.8) containing 1% Tween 80 and transferred into a dialysis bag (dialysis tubing cellulose membrane with an average flat width of 33 mm (1.3 in.), *M*_w_ cut-off (MWCO): 14 000, D9652, Sigma-Aldrich). The dialysis bags were placed into a beaker containing 100 mL of dissolution medium at 37 ± 1 °C. The receptor compartment/beaker was closed to prevent evaporation of the release medium. Samples of the medium (2 mL) were withdrawn and replaced with fresh medium at 1, 2, 3, 6, 9, 12, 24, 48, and 72 h. VAP concentration in the samples was quantified by a UV spectrophotometer (330 nm). The *in vitro* dissolution study was repeated three times for F9-HP and pure HP, then the results were calculated as mean ± SD. The results were then plotted as the cumulative release.

### *In Vitro* Release Kinetics

Release kinetics were investigated using DDSolver software.^46,47^ After obtaining the release profiles, data were transferred to the DDSolver program to determine the four most important and popular criteria: coefficient of determination (Rsqr, *R*^2^, or COD), adjusted coefficient of determination (Rsqr_adj or *R*_adjusted_^2^), Akaike information criterion (AIC), and model selection criterion (MSC). The highest *R*^2^, *R*_adjusted_^2^, and MSC values and the lowest AIC values were used for evaluating Zero-order, Zero-order (*T*_lag_), Zero-order (*F*_0_), First-order, First-order (*T*_lag_), First-order (*F*_max_), First-order (*T*_lag_ and *F*_max_), Higuchi, Higuchi (*T*_lag_), Higuchi (*F*_0_), Korsmeyer–Peppas, Korsmeyer–Peppas (*T*_lag_), Korsmeyer–Peppas (*F*_0_), Hixson–Crowell, Hixson–Crowell (*T*_lag_), Hopfenberg, Hopfenberg (*T*_lag_), Baker–Lonsdale, Baker–Lonsdale (*T*_lag_), Peppas–Sahlin, Peppas–Sahlin 1 (*T*_lag_), Peppas–Sahlin 2, Peppas–Sahlin 2 (*T*_lag_), Quadratic, Quadratic (*T*_lag_), Weibull 1, Weibull 2, Weibull 3, Weibull 4, Logistic 1, Logistic 2, Logistic 3, Gompertz 1, Gompertz 2, Gompertz 3, Gompertz 4, Probit 1, and Probit 2 models.

### Keratinocyte Cell Culture

HaCaT keratinocyte cells were grown in Dulbecco’s Modified Eagle’s Medium (DMEM) at 37 °C in a humid incubator with 5% CO_2_ and 20% fetal bovine serum (FBS), 1% penicillin/streptomycin, 1% l-glutamine, and 1% sodium pyruvate. The cells were passaged and transferred to new flasks, or cell stocks were prepared for use in subsequent experiments once the proliferating cells reached a density of 70–80%. Before the experiments, the cells were counted using the cell counter Cedex XS (Roche-Innovatis, Germany) after being stained with Trypan blue solution to determine the appropriate cell numbers.

### Determination of Concentrations That Increase Cell Proliferation in Keratinocyte Cells

The (3-(4,5-dimethylthiazol-2-yl)-2,5-diphenyltetrazolium bromide) (MTT) assay, which has been used to determine the concentrations that increase cell proliferation, is the result of the succinate dehydrogenase enzymes of the intact mitochondria in living cells destroying the tetrazolium ring in the structure of the dye. It is based on a colorimetric analysis of the formazan salt. Cell viability is directly proportional to the amount of formazan salt present.

HaCaT cells were grown in growth media before seeding at a density of 1 × 10^4^ cells per well in 96-well plates and incubated for 24 h to allow the cells to adhere to the bottom of the plate. Cells were treated for 24 h with concentration groups containing 400, 100, and 10 g/mL VAP, nanoparticle formulation of VAP (NP-1, NP-2, NP-3, NP-4, NP-5, NP-6, NP-7), and control (NP-1-Blank, NP-2-Blank, NP-3-Blank, NP-4-Blank, NP-5-Blank, NP-6-Blank, NP-7-Blank). The existing media in the wells were discarded, and 10 μL of MTT reagent (0.5 mg·mL^–1^) in 100 μL of growth medium was added to the wells. After 3 h, the medium was discarded, 100 μL of dimethyl sulfoxide (DMSO) was added to each well, and absorbance at 540 and 570 nm wavelengths were measured in a Cytation 3 multimode reader device (Biotek). The absorbance values obtained from the wells were correlated to the number of live cells, which was expressed as a percentage of the control group.^48^

### Determination of Wound Healing Effects of Nanoparticle Formulations

To elucidate the wound healing activity, an Oris Cell Migration Assay Kit (Platypus Technologies) was used. HaCaT cells were seeded at a density of 5 × 10^4^ in a 96-well custom plate with a barrier. The barriers were removed from the cells after 24 h with a comb, and the cells were incubated for 24 h in 100 μL of medium containing 10 and 100 μg·mL^–1^ VAP, 10 μg·mL^–1^ NP-3, NP-6, and NP-7, and control. Before analysis, media containing concentration groups were removed, and wells were imaged for wound diameter variation using a Leica DM 300 light microscope (x4). The cells were then labeled with an immunofluorescent dye (Hoechst 33258), and the fluorescence absorption was measured and plotted with the Cytation 3 cell imaging multimode reader.^49^

### Statistical Analysis

Each experiment’s data was entered into GraphPad Prism 7.0 software, and then the replicates were averaged, and standard deviations were determined. Graphics were created using the same program, and the data were statistically evaluated using one-way ANOVA and Tukey’s post hoc test. The data are presented as the means of three independent experiments (*n* = 8 for cell viability assays, *n* = 3 for others), standard deviation (SD), and *p < 0.05, **p < 0.01, ***p < 0.001, and ****p < 0.0001 were judged to be significant when compared to the control group. *p* > 0.05 was considered nonsignificant.

## Results and Discussion

### Particle Size, Polydispersity Index (PDI), ζ-Potential, and Encapsulation Efficiency

PS, PDI, ZP, and EE% analysis results are presented in Table 2. The first thing to notice in particle size analysis is that the VAP-loaded nanoparticles have a larger particle size than blank formulations. When previous studies were examined, it was reported that the particle size of drug-loaded nanoparticle formulations could be larger than blank formulations. The reason for this can be explained by the fact that substances such as polymers encapsulate the active substance and form the particle, and therefore, there may be an increase in particle size.^17,41,50^ Another point that draws attention in particle size analyses is that the particle sizes of chitosan-modified nanoparticles (NP-2, NP-3, NP-4, NP-5, NP-6, NP-7, and their blank formulations) differing from those prepared only with PLGA (Blank-NP-1 and NP-1) were observed to increase. In addition, when the chitosan-modified formulations were examined within themselves, an ordering of particle sizes as NP-2 > NP-3 > NP-4 > NP-5 > NP-6 > NP-7 was observed. The particle size value decreased with the decrease of the chitosan concentration used in the preparation of the formulations. This can be explained by the increase in viscosity associated with chitosan, which reduces the shear stress during the mixing of the emulsion with the magnetic stirrer and subsequently leads to an increase in the particle size of the emulsion droplets.^42^

**Table 2:** Particle Size, PDI, ζ-Potential, and EE % Results

code	particle size (nm, mean ± SD)	PDI (mean ± SD)	ζ-potential (mV, mean ± SD)	EE% (mean ± SD)
blank-NP-1	181.77 ± 2.38	0.12 ± 0.03	– 15.47 ± 0.23	-
NP-1	196.33 ± 0.65	0.11 ± 0.04	–15.60 ± 0.44	74.69 ± 4.04
blank-NP-2	559.63 ± 4.08	0.21 ± 0.02	+39.07 ± 3.04	-
NP-2	669.23 ± 5.49	0.37 ± 0.06	+36.33 ± 0.57	77.86 ± 2.53
blank-NP-3	528.63 ± 2.50	0.16 ± 0.03	+39.67 ± 0.96	-
NP-3	548.43 ± 5.94	0.30 ± 0.04	+30.67 ± 1.10	78.64 ± 6.44
blank-NP-4	506.70 ± 1.56	0.34 ± 0.03	+45.63 ± 0.40	-
NP-4	534.50 ± 3.78	0.37 ± 0.04	+46.13 ± 0.70	81.28 ± 2.80
blank-NP-5	475.97 ± 1.87	0.27 ± 0.01	+47.67 ± 0.51	-
NP-5	480.87 ± 2.70	0.21 ± 0.03	+48.50 ±1.85	85.18 ± 0.58
blank-NP-6	443.40 ± 3.70	0.21 ± 0.05	+52.27 ± 0.9	-
NP-6	469.07 ± 2.37	0.24 ± 0.02	+52.47 ± 1.23	84.78 ± 2.91
blank-NP-7	353.90 ± 6.37	0.15 ± 0.03	+47.03 ± 0.87	-
NP-7	413.60 ± 5.77	0.20 ± 0.01	+45.43 ± 1.34	83.87 ± 2.73

PDI, which gives information about the homogeneity of the particle size distribution in a particular nanosystem, reflects the quality of nanoparticle dispersion in the range of 0.0–1.0. The fact that the PDI value is smaller than 0.1 indicates high dispersion quality and indicates that the particle size is equal to almost all particles. Most researchers accept that PDI values are less than 0.3 in nanoparticle systems prepared as an optimum value; however, values smaller than 0.5 have been reported in many studies in the literature where it is acceptable.^51^ All nanoparticles presented PDI values lower than 0.5 (Table 2), and therefore particle size distribution was decided to be monodisperse.^12^

Negative ζ-potential values were observed in PLGA nanoparticles (Blank-NP-1 and NP-1) not coated with chitosan (Table 2). PLGA in a neutral medium has negative surface potential due to its terminal carboxyl groups, which explains the negative ζ-potential obtained in PLGA nanoparticles uncoated with chitosan.^42^ A colloidal system with a ζ-potential of ±30 mV is considered a stable formulation if it is dispersed as a colloidal dispersion in a liquid.^6,52^ ζ-potential values between −5.0 and −15.0 mV are in the boundary region of flocculation for a nanosystem, and values between −5.0 and −3.0 mV have been previously reported to be the maximum flocculation region for a nanosystem.^53^ When the results for Blank-NP-1 and NP-1 coded formulations are examined, it is seen that the ζ-potential values are slightly above the flocculation limit. This demonstrates the stability of Blank-NP-1 and NP-1 encoded PLGA nanoparticle formulations. ζ-potential values of nanoparticles reached positive values in chitosan-modified formulations (NP-2-7). The positive ζ-potential results obtained are a result of the amino groups found in chitosan and show that the PLGA nanoparticles are adequately covered by chitosan.^7^

The EE% results are presented in Table 2. The high encapsulation efficiency values obtained for both the PLGA nanoparticle and the chitosan-coated PLGA nanoparticles are probably due to the low affinity of VAP to the water phase and thus its lipophilic nature, which tends to migrate to the organic phase.^43^ The content of active ingredients loaded/encapsulated into nanosystems is an important factor in formulations because higher loading allows a lower amount of nanosystems to be used for a given dose. The high encapsulation rates obtained prove that the nanoparticle formulations prepared in the study can be used in lower amounts.

### *In Vitro* Release

**Figure 1:**
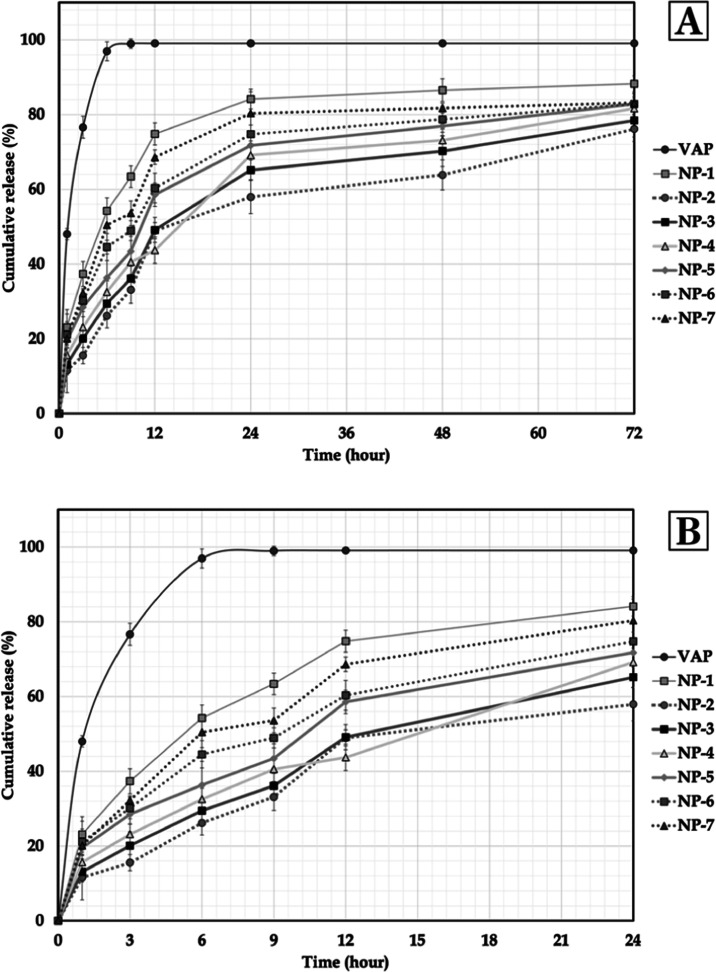
*In vitro* dissolution/release profile of VAP and formulations. *Dissolution medium: (PBS, pH 6.8) containing 1% Tween 80, (A) 72 h profile, (B) 24 h profile.

*In vitro* release profile of pure VAP and formulations are shown in Figure 1. When previous studies with VAP were examined, it was observed that VAP was released more easily in dissolution studies using surfactants.^25,54−58^ In our study, pure VAP showed a release rate of 76.64 ± 2.96% at the end of the 3rd h in PBS medium (pH 6.8) containing 1% Tween 80, and showed a release rate of 96.95 ± 2.56% at the end of the 6th h, showing an almost 100% release rate and was found to be compatible with the literature. Figure 1 shows that it is quite clear that all formulations have prolonged release compared to pure VAP. A rapid release was obtained between the 1st and 6th h (Figure 1). This can be interpreted as VAP being released more rapidly from areas close to the surface of nanoparticle formulations.^56^ The first 24 h release is very important as the formulations are intended for topical use. It can be said that the nanoparticle formulations prepared in line with the results obtained at the end of the 24th h are suitable for topical use. Details of the release studies are discussed in the following paragraph.

At the end of the 3rd and 6th h, the release rates of NP formulations containing VAP are shown in Figure 1. It is quite clear that all formulations produce prolonged dissolution when compared to pure VAP. We interpreted that a rapid dissolution was obtained between the 1st and 6th h, as we mentioned in the above paragraph, as VAP is released faster from the places where they are close to the surface of the NP formulations. It was observed that the VAP release rate from the nanoparticle formulations prepared in almost all time intervals was in the form of NP-1 > NP-7 > NP-6 > NP-5 > NP-4 > NP-3 > NP-2. As a result of not coating the surface of NP-1 with chitosan, a faster release was obtained. For formulations coded NP-2, NP-3, NP-4, NP-5, NP-6, and NP-7, chitosan solution was used in concentrations of 0.2, 0.2, 0.1, 0.05, 0.025, and 0.0125%, respectively. When the chitosan-modified formulations were examined, the release rate decreased inversely with increasing concentrations of the coating solution, and this situation was found to be consistent with the literature.^7^

### *In Vitro* Release Kinetics

After the release rates and cumulative release profiles were obtained, the data were transferred to the DDSolver program to determine the four most important and popular criteria for determining release kinetics. These four criteria were determined as Akaike information criterion (AIC), correlation coefficient (*R*^2^), adjusted correlation coefficient (*R*_adjusted_^2^), and model selection criterion (MSC). The evaluation is based on the highest *R*^2^, *R*_adjusted_^2^, MSC value, and the lowest AIC value.^46,47^ When all of these criteria are taken into consideration, compliance with the Weibull Model was determined in the prepared nanoparticle systems (Table 3). The Weibull model is a very suitable model for matrix-type nanodrug delivery systems.^59−61^ In addition, a high correlation was observed in Korsmeyer–Peppas and Weibull models. It has been previously reported that drug release from NPs may fit more than one model.^39,62^

**Table 3:** *In Vitro* Release Kinetics Results for the Weibull Model and Korsmeyer–Peppas Model

parameter	NP-1	NP-2	NP-3	NP-4	NP-5	NP-6	NP-7
Weibull Model							
Rsqr	0.982	0.972	0.981	0.980	0.979	0.986	0.973
Rsqr_adj	0.975	0.963	0.975	0.974	0.972	0.981	0.964
AIC	50.648	51.109	48.564	49.195	50.079	46.510	53.170
MSC	2.785	2.633	2.979	2.925	2.779	3.118	2.462
β	0.472	0.549	0.535	0.511	0.472	0.453	0.470
Korsmeyer–Peppas Model							
Rsqr	0.894	0.936	0.945	0.964	0.953	0.946	0.891
Rsqr_adj	0.878	0.927	0.937	0.959	0.947	0.939	0.875
AIC	64.423	56.651	56.200	52.659	55.231	56.520	63.825
MSC	1.254	2.017	2.131	2.541	2.207	2.006	1.279
*n*	0.318	0.471	0.442	0.407	0.358	0.335	0.341

Since a high correlation was observed in Korsmeyer–Peppas and Weibull models, some parameters were investigated to further elucidate the release kinetics. Especially in the Korsmeyer–Peppas model, the “*n*” value is the diffusion exponent indicating the drug release mechanism. The *n* value related to the release mechanism can have different values and ranges. These values and ranges can be as follows: *n* < 0.5, *n* = 0.5, 0.5 < *n* < 1.0, *n* = 1, or *n* > 1.0. If *n* < 0.5, the drug delivery system releases by the semi-Fickian diffusion mechanism, if *n* = 0.5, the drug delivery system releases by the Fickian diffusion mechanism, if 0.5 < *n* <1.0, the drug delivery system releases by the anomalous (non-fickian) diffusion mechanism. It has been reported in the literature that if *n*=1, the drug delivery system releases by the non-Fickian state II mechanism, and if *n* > 1.0, the drug delivery system releases by the non-Fickian superstate II mechanism.^63^ The “*n*” value for formulations coded NP-1, NP-2, NP-3, NP-4, NP-5, NP-6, and NP-7 is obtained as 0.318, 0.471, 0.442, 0.407, 0.358, 0.335, and 0.341, respectively (Table 3). In this case, it can be said that VAP release from all nanoparticle formulations prepared in this study is realized by the semi-Fickian diffusion mechanism.

For the Weibull Model, the “β” value is a parameter of the transport mechanism of a drug through the polymeric nanoparticle matrix. Values of β ≤ 0.75 indicate Fickian diffusion, while values of “β” in the range of 0.75–1 indicate the combined mechanism of Fickian diffusion and swelling-controlled release.^64^ The “β” value for formulations coded NP-1, NP-2, NP-3, NP-4, NP-5, NP-6, and NP-7 was obtained as 0.472, 0.549, 0.535, 0.511, 0.472, 0.453, and 0.470, respectively (Table 3). According to the literature, it can be said that VAP release from all nanoparticle formulations prepared in this study is realized by the Fickian diffusion mechanism.

Therefore, to reveal the mechanism of drug release from fabricated NPs, we used mathematical modeling to analyze the *in vitro* release profile of VAP by various kinetic models. A higher correlation was observed in the Weibull model and the Korsmeyer–Peppas model (Table 3). Therefore, our results indicate that the release of VAP NPs is not predominantly driven by a solo mechanism but a combined mechanism of Fickian (pure diffusion phenomenon) and non-Fickian release (due to the relaxation of the polymer chains between the networks).^62^

### Evaluation of Concentrations That Increase Proliferation in HaCaT Cells

**Figure 2:**
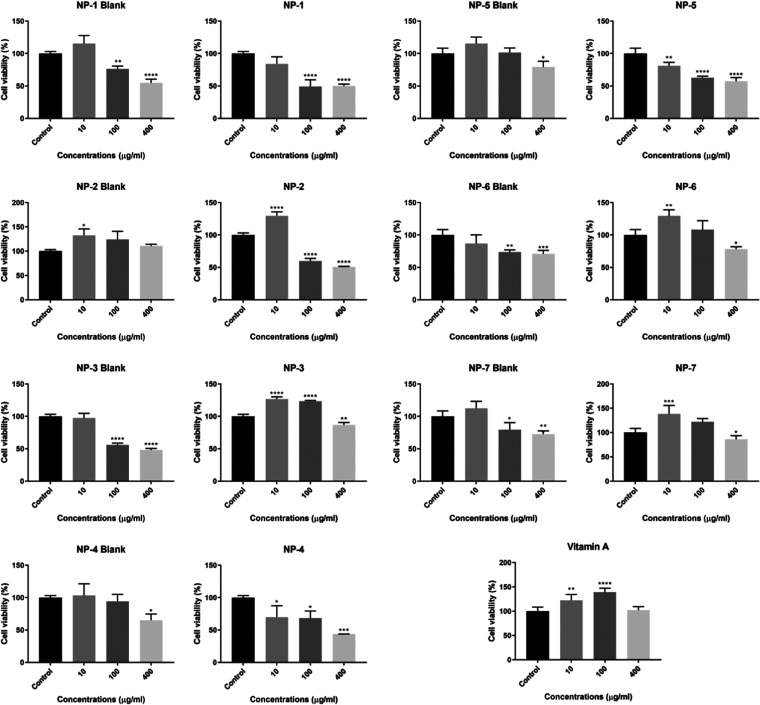
Cell proliferation analysis results of 10, 100, and 400 μg/mL vitamin A, NP-1–7, and NP-1–7 Blank on HaCaT cells. The cell viability results obtained from cells treated with the concentration groups for 24 h were used to generate the proliferation graphs. The results are presented as the mean ± SD of three different experiments, *n* = 8 for each treatment group, significant difference: *p < 0.05, **p < 0.01, ***p < 0.001, ****p < 0.0001; no difference: *p* > 0.05 ns.

A cell proliferation analysis was performed to determine the effective concentrations of keratinocyte proliferation. According to the results of this analysis, vitamin A increased cell viability by 122.59 and 139.14% at 10 and 100 μg·mL^–1^ concentrations, respectively. A total of 10 μg·mL^–1^NP-3, 6, and 7 were found to be effective on cell proliferation, increasing cell proliferation by 126.52, 129.26, and 138.49%, respectively, when compared to vitamin A (Figure 2). According to the findings, nanoparticles, particularly NP-7, have an effect similar to high concentrations of vitamin A (100 μg·mL^–1^) at low concentrations (10 μg·mL^–1^). One notable result is that NP-1-blank outperformed the NP-1 group in promoting cell proliferation. As is known, VAP is a toxic substance; it is known to be a skin irritant and has some side effects such as skin dryness, wounds, and toxicity. The effect on NP-1 can be interpreted as a rapid release of VAP from NP-1.^65^

### Evaluation of Wound Healing Effects of Nanoparticle Formulations Containing Vitamin A Palmitate

Nanoparticle formulations’ impact on wound healing activity was compared to VAP’s. These formulations were found to be effective at stimulating keratinocyte cell proliferation. Plates with a ready-made stopper and facilitating the formation of the identical wound in each well were used for this measurement as an alternative to the scratch test, which is routinely used to evaluate wound healing activity.

**Figure 3:**
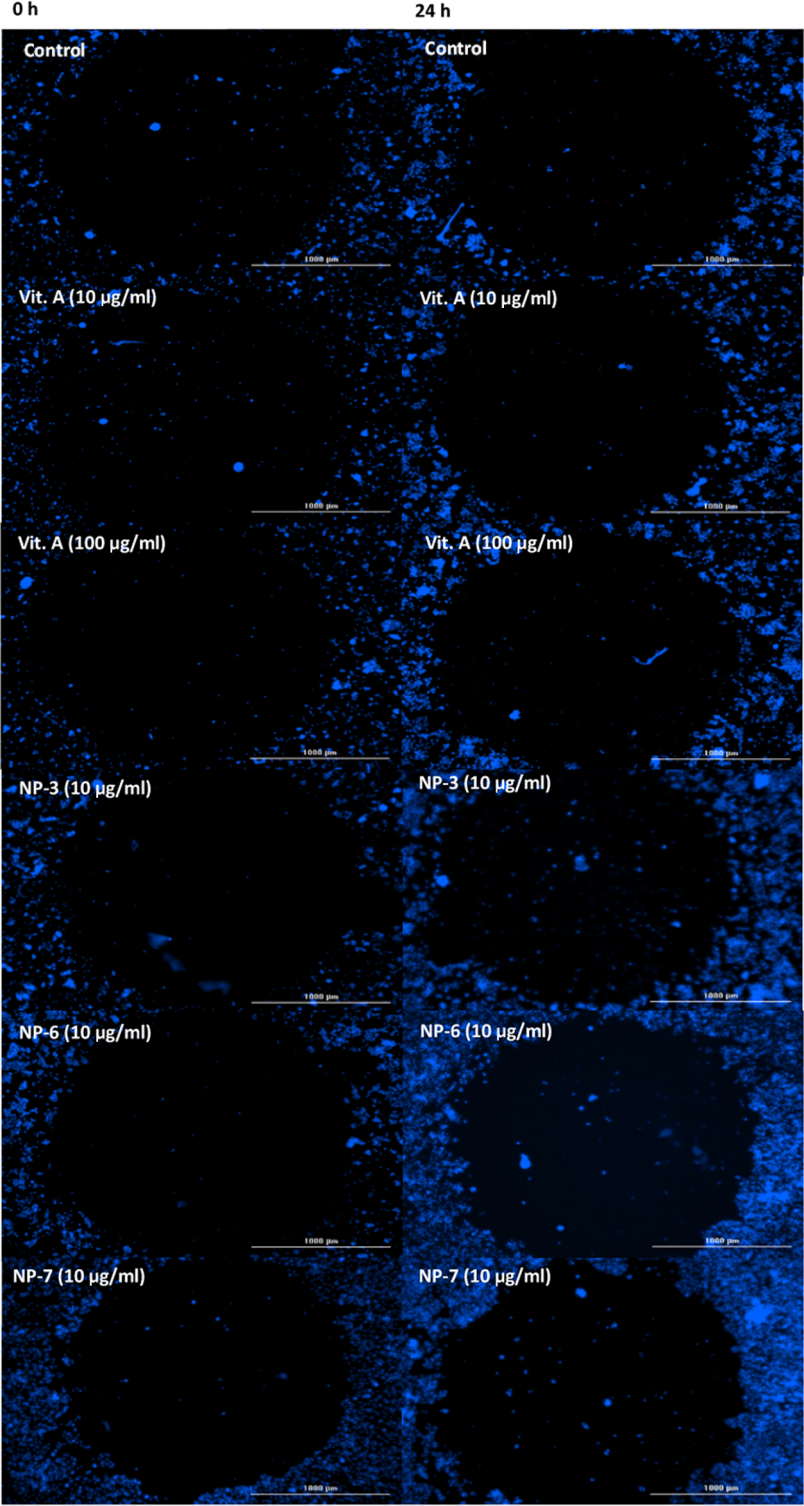
Images demonstrate the wound healing effects of the most potent formulations of vitamin A and vitamin A palmitate nanoparticles (NP-3, 6, and 7) on HaCaT cells at 0 and 24 h (a representative result is shown for each group from two independent replicates, objective ×20).

**Figure 4:**
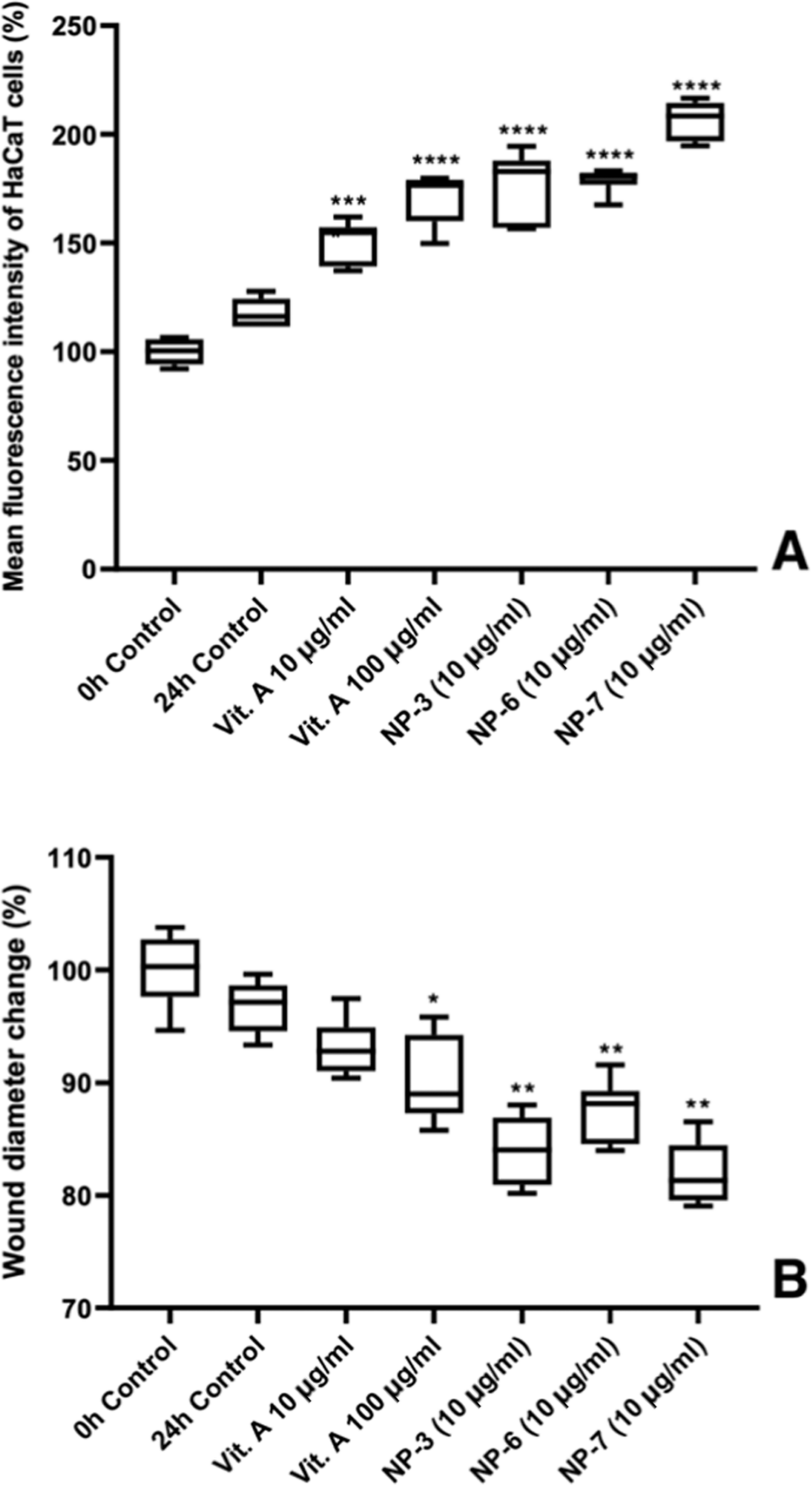
Wound healing (A) and wound diameter change (B) effects on HaCaT cells of the most effective nanoparticle formulations containing Vitamin A palmitate (NP-3, 6, and 7). The box plot represents wound healing with concentration groups at 24 h, relative to the mean fluorescence intensity (A) and wound diameter (B) measured across all groups at hour 0. The data are the mean standard deviations of two different experiments (*n* = 3 for each). One-way analysis of variance (one-way ANOVA) was used to determine the statistical significance of the % values, followed by a post hoc Tukey multiple comparison test (compared to the control group; no difference: *p* > 0.05; significant difference: ****p < 0.0001).

Following the wound formation, 0 h measurements were made, and after 24 h treatment with Vit. A and NPs, the changes in the wound diameter were measured, and cell densities were quantified using fluorescence labeling. According to the results, mean fluorescence intensity expressing cell number change (Figure 3) and percentage values (Table 4), at 24 h, Vit. A 10 and 100 μg/mL NPs 3, 6, and 7 (10 μg/mL) were measured as 150.67, 170.85, 176.59, 179.03, and 206.57%, respectively. The mean fluorescence intensity of the control group at 24 h increased to 117.76% according to the 0 h (100%) (Figure 4A). Wound diameter change results expressing wound closure were found to be 93.13, 90.18, 83.98, 87.50, and 81.94% in the same groups, respectively. The wound diameter of the 24 h control was found to be 96.76% according to the 0 h (100%) (Figure 4B).

**Table 4:** Average Fluorescence Intensity Percentage Values of Wound Healing Effects of Vitamin A and NP-3, 6, 7 Concentrations

0 h control	24 h control	vit. A (10 μg·mL^–1^)	vit. A (100 μg·mL^–1^)	NP-3(10 μg·mL^–1^)	NP-6(10 μg·mL^–1^)	NP-7(10 μg·mL^–1^)
100	117.76	150.67	170.85	176.59	179.03	206.57

Following the wound formation, 0 h measurements were made, and after 24 h treatment with Vit. A and NPs, the changes in the wound diameter were measured, and cell densities were quantified using fluorescence labeling. According to the results, mean fluorescence intensity expressing cell number change, at 24 h, Vit. A 10 and 100 μg/mL NPs 3, 6, and 7 (10 μg/mL) were measured as 150.67, 170.85, 176.59, 179.03, and 206.57%, respectively. The mean fluorescence intensity of the control group at 24 h increased to 117.76% according to the 0 h (100%) (Figure 3A). Wound diameter change results expressing wound closure were found to be 93.13, 90.18, 83.98, 87.50, and 81.94% in the same groups, respectively. The wound diameter of the 24 h control was found to be 96.76% according to the 0 h (100%) (Figure 3B).

## Conclusions

In this study, VAP-loaded PLGA and chitosan-coated PLGA nanoparticles were prepared and characterized. The particle size of VAP-loaded nanoparticles was obtained between 196.33 ± 0.65 and 669.23 ± 5.49 nm. It was determined that the particle size value decreased with the decrease of the chitosan concentration in the formulations in which the chitosan solution was used. PDI data proved that all nanoparticles were prepared as high quality and monodisperse. While negative ζ-potential values of Blank-NP-1 and NP-1 encoded PLGA nanoparticle formulations were obtained, positive ζ-potential was obtained in other nanoparticles. These positive ζ-potential results were a result of the amino groups found in chitosan. Due to the low affinity of VAP to the water phase, high encapsulation efficiency has been achieved, and the encapsulation efficiency has been achieved in the range of 74.69–85.18%. *In vitro* dissolution studies of nanoparticles observed rapid dissolution in the first 1–6 h but prolonged dissolution of VAP after rapid dissolution. The dissolution kinetics are predominantly governed not only by a single mechanism but also by a combined Fickian and non-Fickian mechanism. As a result of cell culture studies and wound healing activity studies, it was determined that NP-7 was the most effective. It was thought that the reason for this was that the NP-7 coded formulation was a chitosan-coated PLGA nanoparticle with the smallest particle size, and it was concluded that the efficiency of VAP was increased with its nanoparticle structure. As a result, low-dose high wound healing activity was found with the VAP-loaded nanoparticle structure. Since the prepared nanoparticles are intended for cosmetic and topical use, the addition of the NP-7 coded formulation to a semi-solid carrier system, characterization, and efficacy studies are planned in the next stages of the study.
